# Modulation of Signaling Mediated by TSLP and IL-7 in Inflammation, Autoimmune Diseases, and Cancer

**DOI:** 10.3389/fimmu.2020.01557

**Published:** 2020-07-21

**Authors:** Iva Marković, Savvas N. Savvides

**Affiliations:** ^1^VIB-UGent Center for Inflammation Research, Ghent, Belgium; ^2^Unit for Structural Biology, Department of Biochemistry and Microbiology, Ghent University, Ghent, Belgium

**Keywords:** cytokines, antagonist, agonist, protein-protein complex, therapeutic biologics, cytokine-receptor complex

## Abstract

Thymic Stromal Lymphopoietin (TSLP) and Interleukin-7 (IL-7) are widely studied cytokines within distinct branches of immunology. On one hand, TSLP is crucially important for mediating type 2 immunity at barrier surfaces and has been linked to widespread allergic and inflammatory diseases of the airways, skin, and gut. On the other hand, IL-7 operates at the foundations of T-cell and innate lymphoid cell (ILC) development and homeostasis and has been associated with cancer. Yet, TSLP and IL-7 are united by key commonalities in their structure and the structural basis of the receptor assemblies they mediate to initiate cellular signaling, in particular their cross-utilization of IL-7Rα. As therapeutic targeting of TSLP and IL-7 via diverse approaches is reaching advanced stages and in light of the plethora of mechanistic and structural data on receptor signaling mediated by the two cytokines, the time is ripe to provide integrated views of such knowledge. Here, we first discuss the major pathophysiological roles of TSLP and IL-7 in autoimmune diseases, inflammation and cancer. Subsequently, we curate structural and mechanistic knowledge about receptor assemblies mediated by the two cytokines. Finally, we review therapeutic avenues targeting TSLP and IL-7 signaling. We envision that such integrated view of the mechanism, structure, and modulation of signaling assemblies mediated by TSLP and IL-7 will enhance and fine-tune the development of more effective and selective approaches to further interrogate the role of TSLP and IL-7 in physiology and disease.

## TSLP—Emerging Role in Autoimmune Diseases and Cancer

Over the last two decades TSLP has been extensively studied and known for its pivotal role in allergic conditions and involvement in chronic inflammatory diseases such as chronic obstructive pulmonary disease or inflammatory bowel disease (1–3). In recent years TSLP has additionally emerged as a novel molecular player in non-allergen induced conditions (4, 5). Together with broadening its pathophysiological profile these findings imply that the microenvironment of this pleiotropic cytokine might define the direction of its inflammatory response depending on the type of inflammation involved.

The development of IL-23-associated autoimmune disease psoriasis has recently been linked to overexpression of TSLP in keratinocytes from patient skin samples. It has also been demonstrated that serum levels of TSLP correlate to the severity of the disease (6, 7). Even though the role of TSLP in psoriasis is not completely resolved, TSLP has been reported to induce DC maturation and to drive to a DC-derived IL-23 production leading to the hypothesis that it could have a comparable role in other IL-23-driven autoimmune diseases (8). Furthermore, TSLP has been linked to rheumatoid arthritis (RA): increased TSLP levels in synovial fluid of patients with RA in comparison to those with osteoarthritis have been reported in several studies (9). Moreover, TSLP receptor (TSLPR) has also been found overexpressed in myeloid dendritic cells (mDCs) in synovial fluid of the RA patients. The engagement of TSLP in inflammatory arthritis is explained through TSLP-mediated priming of the mDCs and subsequent chemokine stimulation of CD4+ T cells proliferation leading to secretion of interferon γ (IFNγ), IL-17, and IL-4 (10). Additionally, several reports based on studies via mouse models and in humans provide further evidence of TSLP's possible involvement in the pathogenesis of different types of autoimmune disorders underlining a rising need to further interrogate the role of TSLP in relation to Th17 inflammatory response (11–13).

Thus, given such emerging evidence of the involvement of TSLP in the abrogation of Th1, Th9, and Th17 inflammatory responses and its influence on a range of immune cell lineages it does not come as a surprise that TSLP is becoming more intensively studied in the context of cancer (5, 8, 14). Nevertheless, the current view of the field regarding the role of TSLP in cancer has been divided in terms of tumor-progressive or tumor-protective effects depending on the type of cancer being studied. Studies focusing on tumors of hematopoietic and lymphoid tissues such as lymphoma and acute lymphocytic leukemia (ALL) reported TSLP as a tumor-progressive factor (15, 16). In addition, various genomic analyses detected chromosomal rearrangements and alterations of genes encoding TSLPR/CRLF2 in a large number of patients with B-cell precursor ALL (BCP-ALL), all of them having in common either an enhanced or constitutive expression of TSLPR leading to a signal boost resulting in resistance to therapy, high recurrence rate, and poor clinical outcome (17–19). Interestingly, TSLPR was also found overexpressed in 15% of B-ALL cases with no typical chromosome aberrations (20). Results from diverse experimental and clinical studies in diverse solid tumors—cervical, ovarian, pancreatic, or gastric cancer further imply an evident tumor-progressive role of TSLP in tumor microenvironment leading to the promotion of tumor angiogenesis and its growth and metastasis (21–27). On the other hand, both tumor-progressing and anti-tumor effects of TSLP have been demonstrated in diverse breast cancer studies (28–31). In contrast, skin and colon cancer studies reported TSLP-mediated anti-tumorigenic role hence emphasizing the urgency and importance of understanding this duality in the framework of developing suitable future therapeutics (32, 33).

## Genetic Variations in IL-7 Axis Play a Role in Both Autoimmunity and Cancer

Given that tight control of signaling mediated by IL-7 is essential to support and maintain immune homeostasis, it is not surprising that dysregulation of its stimulation leads to a disrupted lymphoid development and pathophysiology in different types of conditions including autoimmune diseases and cancer. Seeing that the absence of IL-7 mediated signaling leads to lymphopenia, a role of its regulation in autoimmune diseases could be implied (34). Several studies involving patients with multiple sclerosis (MS) and primary Sjögren's syndrome (pSS) demonstrate that both IL-7 and IL-7R are overexpressed in the cerebrospinal fluid and labial salivary glands, respectively, with these expression levels correlating to the severity of the disease (35–37). Increased susceptibility to autoimmune diseases such as MS, type 1 diabetes or RA have been linked to several single nucleotide polymorphisms in IL-7R gene loci (35, 38–40). In the case of MS, haplotypes in *IL7R* gene have been reported to lead to modulations in levels of soluble IL-7R which has also been upregulated in patients with pSS (41, 42). Based on additional extensive data from mice and human *in vitro* experiments, the pivotal involvement of IL-7 is more than evident. However, the mechanism of how the regulation of the IL-7 axis leads to increased susceptibility to autoimmune diseases still remains largely unclear, although the current mechanistic view suggests that activation of IL-7 signaling promotes the expansion of T cells and increased proliferation to self-antigens leading to predisposition to autoimmunity (43).

Whereas, IL-7 signaling is not as crucial in B-cell development in humans as it is in mice, cells from acute leukemia proliferate in response to IL-7 *in vitro* and have a corresponding expression of IL-7Rα (44). In addition, overexpression of IL-7Rα has recently been linked with relapse in pediatric B-ALL (45). IL-7Rα gene loci have been shown to carry gain-of-function mutations in a small fraction of patients with BCP-ALL with most of the mutations being associated with concurrent upregulation of TSLPR upregulation and ligand-independent activation of signaling (46). Involvement of the IL-7 signaling axis in the progression of T-ALL has been confirmed in extensive diverse studies showing stimulation of T-ALL cells with IL-7 and overexpression of IL-7Rα (47–52). This evidence is supported by identification of additional IL-7Rα gain-of-function mutations in T-ALL patients that lead to constitutive IL-7 independent receptor activation or else increased activation of the receptor resulting in increased IL-7 response (53, 54). Taking into account that 10% of T-ALL patients carry *IL-7R*α mutations that have been linked to poor prognosis and risk in relapsed patients, IL-7Rα and its signaling pathways have emerged as logical therapeutic targets (55). Aside from strong evidence that IL-7 has anti-tumor effects, some studies indicate that IL-7 might enhance tumor-progression, for instance as suggested in studies focusing on non-small cell lung cancer cells (56). It is clear that further studies will be needed to better clarify the role of IL-7 in tumor-progression.

## Biology, Receptor Activation, and Signaling of TSLP and IL-7

In response to pathogenic stimuli or mechanic injury, TSLP gets produced mainly by epithelial cells at barrier surfaces such as lung and gut, epidermal keratinocytes, and dendritic cells. However, we now know that TSLP has a much broader expression profile that extends to fibroblasts, macrophages, basophils, monocytes, and cancer cells (57–68). In addition to being involved in proliferation and differentiation of B-cell progenitors, TSLP expression and signaling leads to activation of immature DCs, CD4+ T-cell homeostasis and regulatory T-cell development through a coordinated cascade of immune and non-immune cells indicating its influence far beyond the widely acknowledged type 2 inflammatory responses (5, 61, 69–78).

**Figure 1:**
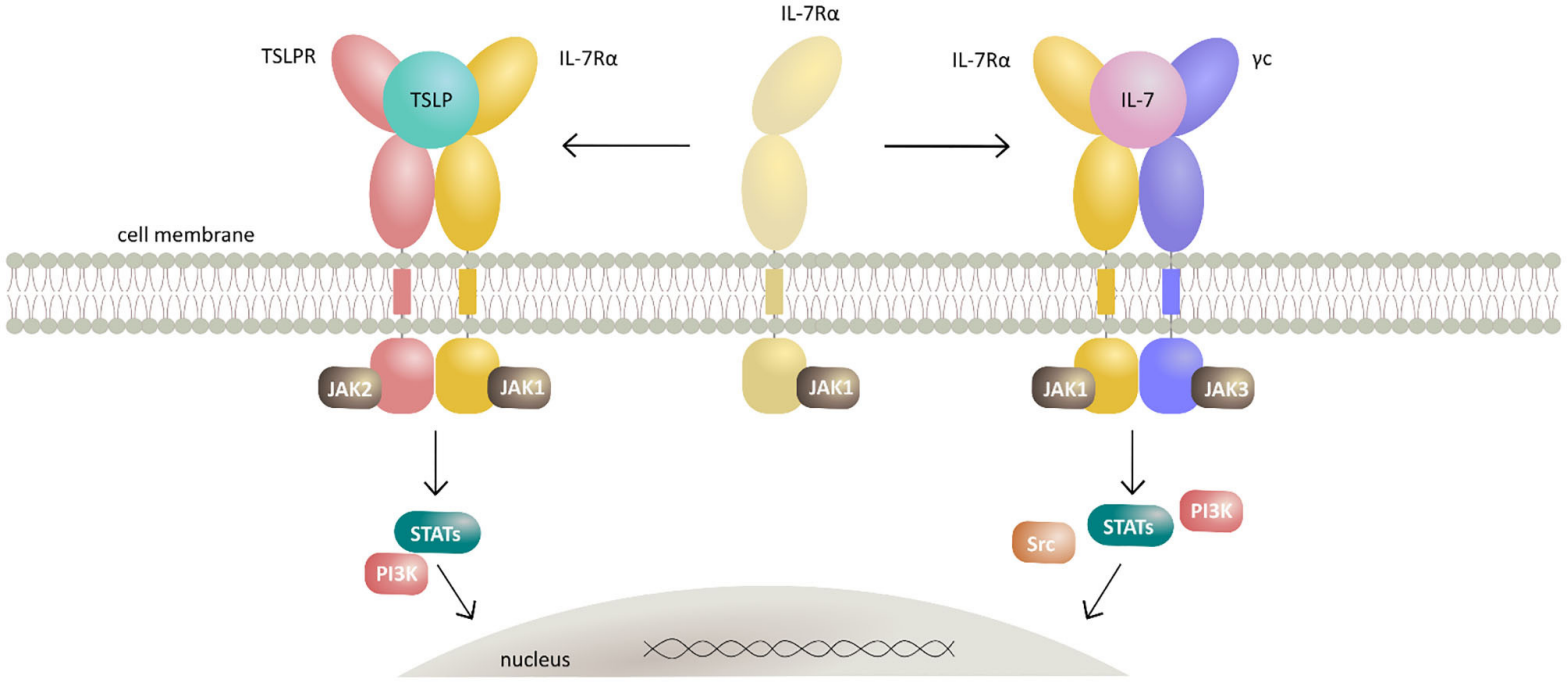
Schematic representation of TSLP and IL-7 signaling mechanisms by their respective receptor heterodimerization upon cytokine binding. Cytokines TSLP and IL-7 both signal through heterodimeric receptors by sharing the IL-7Rα receptor chain. TSLP first interacts with the cognate TSLPR thus potentiating the recruitment of IL-7Rα and formation of extracellular ternary complex leading to activation of intracellular signaling by canonical JAK/STAT and PI3K pathways. Together with IL-7Rα and the γc, IL-7 forms a heterodimeric receptor complex resulting in activation of JAK/STAT, PI3K, and SRC pathways.

TSLP mediates signaling by establishing a heteromeric complex involving TSLPR, a type I cytokine receptor, and IL-7Rα, a receptor also utilized by IL-7 (79, 80). TSLPR binds TSLP with high affinity (*K*_D_ = 32 nM), while IL-7Rα does not bind to TSLPR alone with measurable affinity. However, IL-7Rα can be recruited with high affinity (*K*_D_ = 29 nM) to the TSLP:TSLPR complex making this binary assembly a mechanistic prerequisite for effective signal transduction (81, 82). Of note, human TSLPR and IL-7Rα were shown to bear low, albeit measurable affinity (*K*_D_ 20 μM) for each other in the absence of TSLP, suggesting that preformed receptor-receptor interactions might play a role in the assembly of a TSLP-mediated complex under certain conditions (82). The ensuing dimerization of both receptor chains upon TSLP binding results in activation of Janus kinases (JAKs) and signal transducers and activators of transcription (STATs) leading to transcription of target genes and subsequent tightly coordinated immune responses (83–86) (Figure 1). Recently, tools have been developed exploiting the signaling properties of TSLPR for the screening and characterization of the activity of various types of cytokines and their receptors (87).

Similar to TSLP, IL-7 is predominantly secreted by non-lymphoid cells like keratinocytes and epithelial and stromal cells in lymphoid organs with highest expression levels being detected in thymus and lymph nodes. In contrast to several other members of IL-2 family cytokines, IL-7 is not produced by hematopoietic cells (88–93). In fact, it is now known that together with ILCs, hematopoietic cells express IL-7Rα and therefore play a role in lymphoid consumption and regulation of IL-7 availability (94). While the IL-7Rα on lymphoid cells regulates both TSLP and IL-7-mediated signaling, non-lymphoid cell types carrying IL-7Rα mediate only TSLP signaling (95). IL-7 signaling is essential for the development and homeostasis of T lymphocytes and several members of recently discovered ILC family, whereas its role in early development of B lymphocytes has been shown to be more substantial in mice than in humans (95–97).

IL-7 signals through a heterodimeric receptor complex consisting of IL-7Rα and the γc (98). The observed stepwise mechanism of the ternary assembly mediated by IL-7 is analogous to the one suggested for TSLP and γc family interleukins, whereby the formation of a high affinity IL-7:IL-7Rα constitutes a mechanistic requirement for the assembly of the signaling-competent ternary complex (Figure 1).

However, contrary to TSLP binding to TSLPR, binding of IL-7 to IL-7Rα has been proposed to proceed via biphasic binding kinetics manifested by two sets of on- and off-rate constants (99). The presence of such an unusual IL-7:IL-7Rα structural intermediate implied by the proposed model requires further confirmation by orthogonal biophysical approaches that may allow detection of conformational changes. Additional mechanistic considerations centering on the possibility of IL-7Rα homodimers and IL-7Rα-γc heterodimers in the absence of IL-7, have also been proposed: upon the presence of IL-7 a pre-associated IL-7Rα-γc heterodimer undergoes a rotation away from the cell surface bringing the C-termini of the IL-7Rα and γc within the distance allowing them to form an activating complex (100). A similar mechanism involving an IL-7Rα homodimer could serve as an explanation of the signaling effects that the gain-of-function mutations in T-ALL and B-ALL patients have independent of γc and IL-7 presence (46, 53). As an example, it has been shown that the S185C IL-7Rα mutation present in B-ALL patients leads to the formation of an additional disulfide bond between the two S185C IL-7Rα chains whereas the mutation of the cysteine to a glycine eliminates this effect (46). Results obtained from structural modeling of this interaction confirm the suggested mechanism by showing that the disulfide bond between the receptor chains allows the signal inducing proximity without the presence of a ligand (100).

Strikingly, whereas binding of IL-7 to non-glycosylated IL-7Rα shows medium affinity (low μM range) there is a dramatic decrease in the apparent *K*_D_ when binding the glycosylated IL-7Rα (low nM range) irrespective of glycan type or branching (99). Decoy IL-7Rα also plays an important role in regulation of IL-7 in immunity and in disease states (101). While the membrane bound IL-7Rα chain participates in signaling, soluble IL-7Rα competes with the membrane-bound receptor to eliminate excessive IL-7 thereby re-establishing its low levels as normally observed *in vivo* (102). Ligand-induced dimerization of IL-7 receptor leads to the activation of JAK-STAT, PI3-kinase, and MAPK/Erk signaling cascades and their respective responses (103–108).

## Structural Basis of TSLP-Mediated Signaling

Consistent with its annotation as a member of the IL-2 family of helical cytokines, structural studies by X-ray crystallography of human and mouse TSLP in complex with its cognate receptors revealed that TSLP adopts a four-helix bundle structure with alpha helices (αA, αB, αC, and αD) following an ‘up-up-down-down’ topology and six cysteine residues forming pairs of disulfide bridges (81, 82). Recently, evidence of two isoforms of human TSLP originating from the TSLP gene—a short and a long form—has been reported largely based on mRNA expression profiles (109). Given that it is currently unclear whether the short isoform of TSLP is translated into a biologically active protein, we will only be focusing on the well-established and biologically active long form of TSLP.

**Figure 2:**
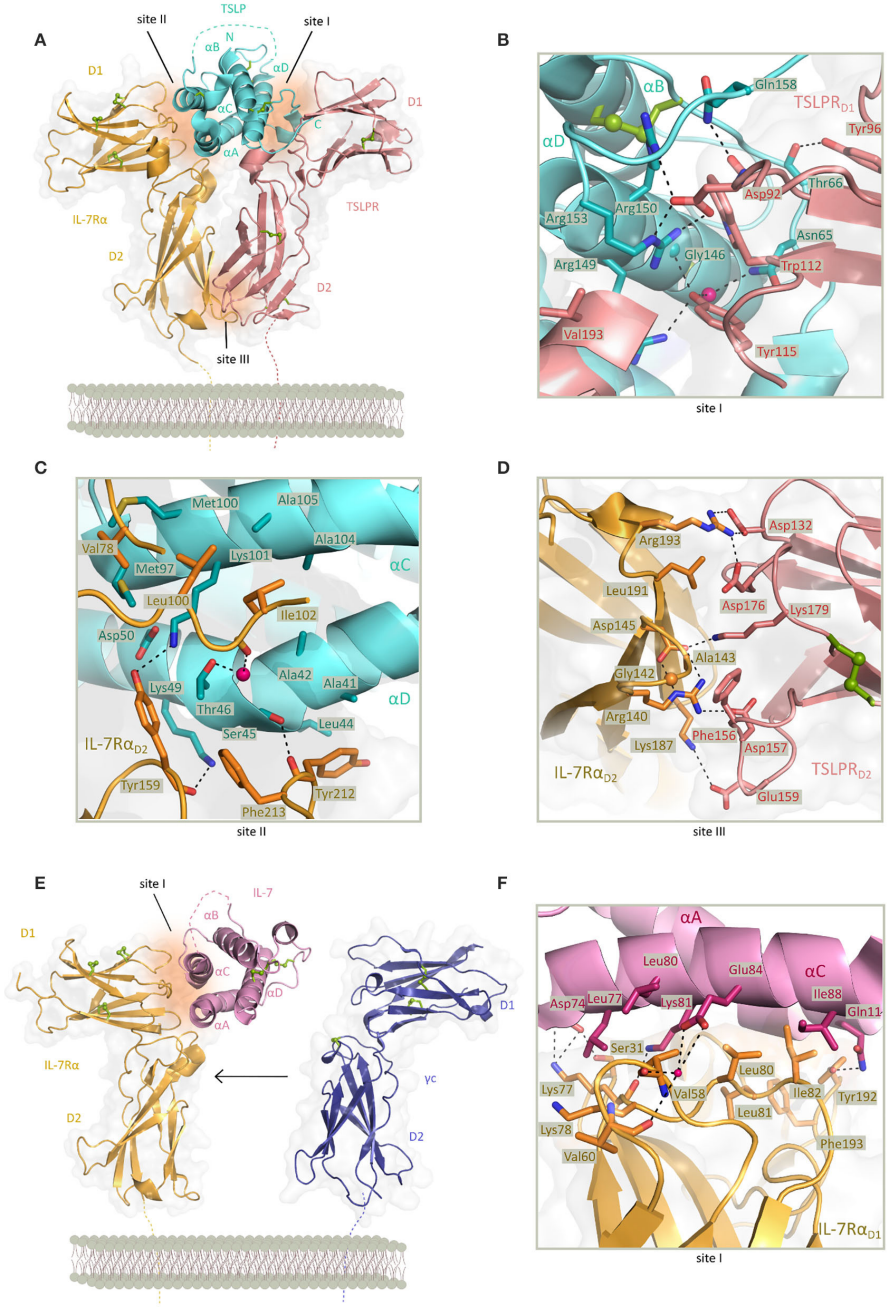
Structure of TSLP and IL-7 receptor complexes and structural close up view of the cytokine-receptor contact interfaces. **(A)** View of the determined X-ray structure for the TSLP:TSLPR:IL-7Rα ternary complex. TSLP is shown in aquamarine cartoon representation with four helices marked αA-αD and the disordered CD loop region is shown as a dashed aquamarine line. The extracellular regions of TSLPR (salmon pink) and IL-7Rα (bright orange) each comprising of two FnIII-like domains D1 and D2 are shown as cartoons on a transparent gray surface representation. Disulfide bridges are represented by green spheres. Regions contributing to protein-protein contact are named site I (TSLP:TSLPR), site II (TSLP:IL-7Rα), and site III (TSLPR:IL-7Rα) and represented by dark orange surfaces. [PDB 5J11, (82)] **(B)** Detailed representation of the TSLP:TSLPR interface (site I). **(C)** Detailed representation of the TSLP:IL-7Rα interface (site II). **(D)** Detailed representation of the TSLPR:IL-7Rα interface (site III) viewed from the membrane-proximal side. In **(B–D)** interface residues are shown as sticks and hydrogen bonds and salt bridges are indicated with a dashed line. Water molecule is depicted as a pink sphere. **(E)** View of the X-ray structure for the IL:IL-7Rα binary complex. IL-7 is shown in pink cartoon representation with four helices marked αA-αD and the second crossover loop shown as a dashed pink line. The extracellular regions of IL-7Rα (bright orange) and γc (purple) are comprising of two FnIII-like domains D1 and D2 shown as cartoons on a transparent gray surface representation. The γc from the IL4 ternary complex has been depicted apart from the IL:IL-7Rα complex as it has not been co-crystallized with the binary complex [PDB 3DI2, 3BPL, (99, 111)]. Disulfide bridges are represented by green spheres. Region contributing to IL-7:IL-7Rα contact is named site I and represented by dark orange surface. **(F)** Detailed representation of the IL-7:IL-7Rα interface (site I). Interface residues are shown as sticks and hydrogen bonds and salt bridges are indicated with a dashed line. Water molecule is depicted as a pink sphere.

Intriguingly, human TSLP displays a unique structure among four-helix bundle cytokines manifested via a rather open helix-bundle core harboring a substantial internal void volume adjacent to a fully buried water molecule coordinated by three conserved residues (Trp148, Thr102, and Thr83). Furthermore, helix αA displays a substantial kink centered at about its midpoint that is hallmarked by a π-helical turn, a structural feature that has been linked to the enhancement of protein functionality (110). Complementary structural studies on unliganded human TSLP in solution by nuclear magnetic resonance established the flexibility of helix αA at its π-helical turn (82). The TSLP four-helix bundle is threaded by three loops, a *BC-, AB-*, and *CD-* loop, with the latter harboring a stretch of 7 basic amino acids (residues 125–131) the role of which remains unclear. However, it has been proposed that this segment encodes for a furin cleavage site and might provide a level of regulation for the secreted amounts of TSLP (82) (Figure 2A).

Based on high-resolution structural insights from the TSLP:TSLPR:IL-7Rα ternary complex, the attraction of TSLP to a cytokine binding homology region (CHR) in TSLPR (site I) is characterized by electrostatic complementarity between a positively charged surface on TSLP and negatively charged one on TSLPR (Figure 2B). Formed binary complex TSLP:TSLPR has a calculated negative electrostatic potential which supports subsequent binding of IL-7Rα having a positive electrostatic potential. Part of residues involved in TSLP:TSLPR interactions are located in the C-terminal part of αD helix (residues 142–158) and *AB*-loop region (residues 60–69) undergoing significant conformational changes for obtaining the bound state. The *AB*-loop is simultaneously offering a physical link to the αA helix important for interactions with IL-7Rα (site II) thus being a mediator of priming TSLP to recruit the IL-7Rα after forming TSLP:TSLPR complex which then facilitates the positioning of the αA helix providing a necessary entropic advantage for the formation of a T-shaped ternary complex. Besides residues in αA helix, hydrophobic interface of IL-7Rα furthermore interacts with several exposed residues in αC helix of TSLP (Figure 2C). Compact interaction of TSLPR and IL-7Rα in their membrane proximal region (site III), also known as the stem region, is characterized by electrostatic interactions and close van der Waals contacts. Interactions in site III have been proven to contribute to the effective TSLP-mediated signal transduction (82) (Figure 2D).

## Structural Basis of Signaling Assemblies Mediated by IL-7

Just like TSLP and other cytokines of the IL-2 family, IL-7 has four helices (αA-αD) adopting the “up-up-down-down” topology. While homology models had predicted the presence of three disulfide bonds, electron density of crystal structures obtained was too weak at the N-terminus and the end of helix αC where third disulfide bond was predicted resulting in tracing only two of the cysteine pairs (99, 112). Structural data shows burial of the only tryptophan residue of IL-7 in the hydrophobic core of the helix bundle, consistent with mutagenesis studies that linked this position to the proper folding of IL-7 (99, 113, 114). As in TSLP, helix αA of IL-7 comprises a π-helical turn of six residues stabilizing the IL-7:IL-7Rα interaction. This interface also includes contacts with αC residues and is generally characterized by hydrophobic, van der Waals and few intermolecular polar interactions (Figures 2E,F).

While other cytokines that signal via γc family receptors interact with their specific receptor chain via a larger buried surface consisting of predominantly polar residues, the IL-7:IL-7Rα interface is not only less extensive but also more hydrophobic (99). Interestingly, the TSLP-bound conformation of IL-7Rα is highly similar to the one observed in IL-7:IL-7Rα and IL-7Rα employs a nearly identical set of residues to bind each of the cytokines. These unique structural features shared between TSLP and IL-7 allow a predominantly hydrophobic interaction with IL-7Rα providing a rationale for duality and degeneracy of signaling via IL-7Rα (82). Nevertheless, the structure of the IL-7:IL-7Rα complex does readily explain the large differences in the affinity of IL-7 to the glycosylated and non-glycosylated forms of IL-7Rα as all candidate glycosylation sites are not in close proximity to the IL7:IL7Rα interface (99).

So far, there has been no structural data for the ternary IL-7:IL-7Rα:γc assembly, which would fill a large void in our understanding of the extracellular signaling assembly mediated by IL-7. Besides comparing structural and mutagenesis data coming from other cytokine-receptor binary and ternary assemblies in γc receptor family, efforts have been made to model this interaction using the IL-7:IL-7Rα binary complex and γc structures extracted from other complexes. These approaches suggested the critical involvement of a disulfide bond and involvement of several key residues on γc for the formation of a ternary complex. Although the models propose the canonical engagement of helices αA and αD in binding the γc receptor, the proposed models also suggest the IL-7: γc interface will differ substantially from other γc:interleukin interactions. The differentiation of cytokines by γc is additionally facilitated by angular displacement of the helices to form a distinct binding epitope. Indeed, superpositon of IL-2, IL-4, and IL-21 as observed in their receptor complexes onto IL-7 revealed a substantial difference in the inter-helical orientation (99). Superpositions of fibronectin type III (FnIII) domains of IL-7Rα and IL-4Rα binary and ternary complexes onto IL-2Rβ show differences in angular geometry between the domains resulting in steric clashes and a lack of availability of helices to contact γc receptor. This suggests that it requires another conformation and a more drastic elbow angle between the two FnIII domains to adequately form IL7:IL-7Rα:γc (99, 100).

## Strategies to Modulate Signaling Mediated by TSLP and IL-7

**Figure 3:**
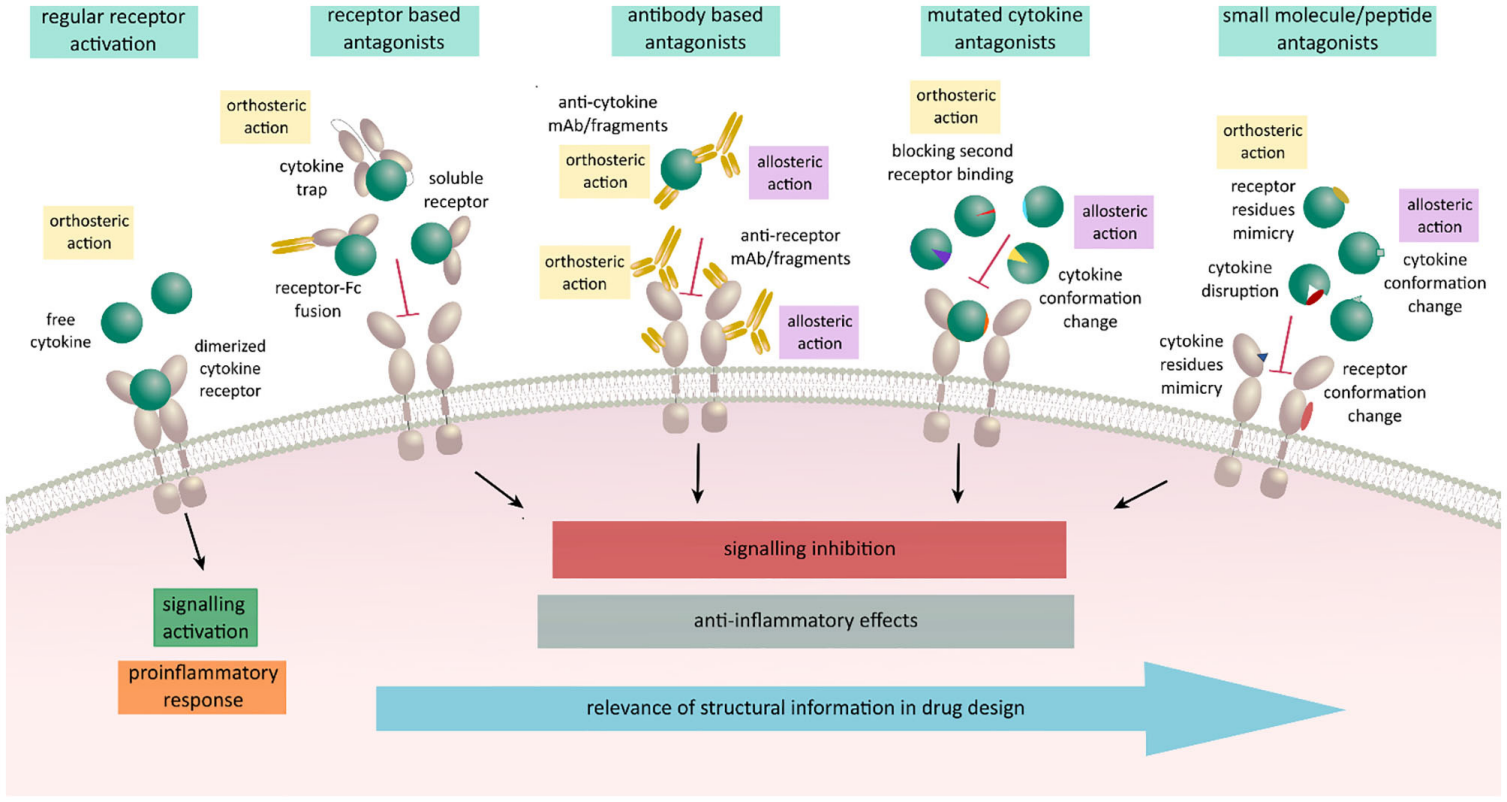
Schematic representation on various antagonistic strategies used for regulation of cytokine signaling acting in an allosteric or an orthosteric fashion. Receptor activation leads to signaling activation upon cytokine binding and receptor dimerization resulting in a proinflammatory responses and various disease states. These responses can be meliorated by the use of biologicals engineered to orthosterically or allosterically block the receptor activation. Receptor based antagonists act in an orthosteric fashion by blocking the expressed cytokine and preventing it to bind to its respective ligand. Antibody based antagonists consist of either full antibodies, Fab fragments, scFv, or nanobodies against the cytokine or its receptor. They can either act orthosterically at the binding site and prevent the binding of cytokine to its specific receptor or allosterically by binding outside of the binding site affecting the conformation and resulting in the lack of binding ability of either cytokine or the receptor. Mutated variants of cytokines are designed to act in both ways by keeping the ability to bind to their specific cytokine receptor chain and losing the affinity to the second receptor chain due to selected mutations. Small molecule and peptide antagonists design is based on either mimicking cytokine or receptor interaction residues, disrupting the proper folding of the protein or binding outside of the interaction sites inducing a conformation change resulting in blocking of receptor activation and signal transduction.

In light of the tremendous importance of cytokines in health and disease, recent efforts have focused on harnessing structural and functional data interrogating cytokine-receptor interactions and functionality toward the development of potent antagonists and agonists that can modulate cytokine-mediated signaling (115) (Figure 3). Such modulators would be expected to have distinct modes of action compared to already available inhibitors known to target intracellular portions of cytokine receptors or specific intracellular signaling components downstream of cytokine-dependent receptor activation (116, 117). Nevertheless, extracellular portions of cytokine receptors and the activating cytokines are attractive targets in their own right. In this context, modulation of their activity could be achieved by both biologicals and non-biologicals engineered to bind in either orthosteric or allosteric fashion. Preventing cytokine action in such a context has mostly been achieved by neutralizing antibodies developed against either the cytokine or one of its receptors or by using soluble ectodomains of the cytokine receptors as molecular decoys (101, 118–121). These approaches do not necessarily require structural insights and are therefore favorable in targeting cytokine/receptor interactions when no such data is available. To this end, promising alternatives are in the form of Fc-fused receptors or cytokine traps comprising fusions of the cognate receptor ectodomains via flexible linkers (82, 122–124). Cytokine-derived antagonists present another approach in which structural information together with the mechanistic insights are crucial for establishing the critical cytokine-receptor interaction determinants to arrive at cytokine variants having desired modulatory characteristics and potency (115). In the case of heterodimeric receptor complexes, this approach is based on the engineering a cytokine such that it can only bind one of the receptor chains with high affinity thereby being unable to recruit the second receptor to form a signaling complex with IL-4 antagonist as one of the best-known examples (113, 125). Such a strategy led to the design of muteins with remarkable new properties: for instance, an IL-4 antagonist (Pitrakinra™) is able to block both IL4/IL-13 receptor assembly formation, an IL-2 superkine where the engineered new property bestows a higher affinity for IL-2Rβ chain, or the most recently reported neoleukins designed to play into the functional dichotomy of IL-2 and IL-15 (126–128).

Peptide-based inhibitors can have high structural similarity to fragments of the target proteins and in that way mimic protein-protein interactions crucial for signaling. Their additional advantage is simple synthesis and possibility to modify their peptide sequences using diverse functional groups (129, 130). Together with small molecule inhibitors, such inhibitors focus on targeting hot spots and binding gaps at cytokine-receptor interfaces and modulate their activity as demonstrated for IL-2 and TNF and suggested for IL-18 (131–134). Although challenging to develop, potent small molecules inhibitors are considered to offer substantial advantages over biologics or protein-based modulators, including oral and topical administration (135).

## Modulators of TSLP Signaling

**Figure 4:**
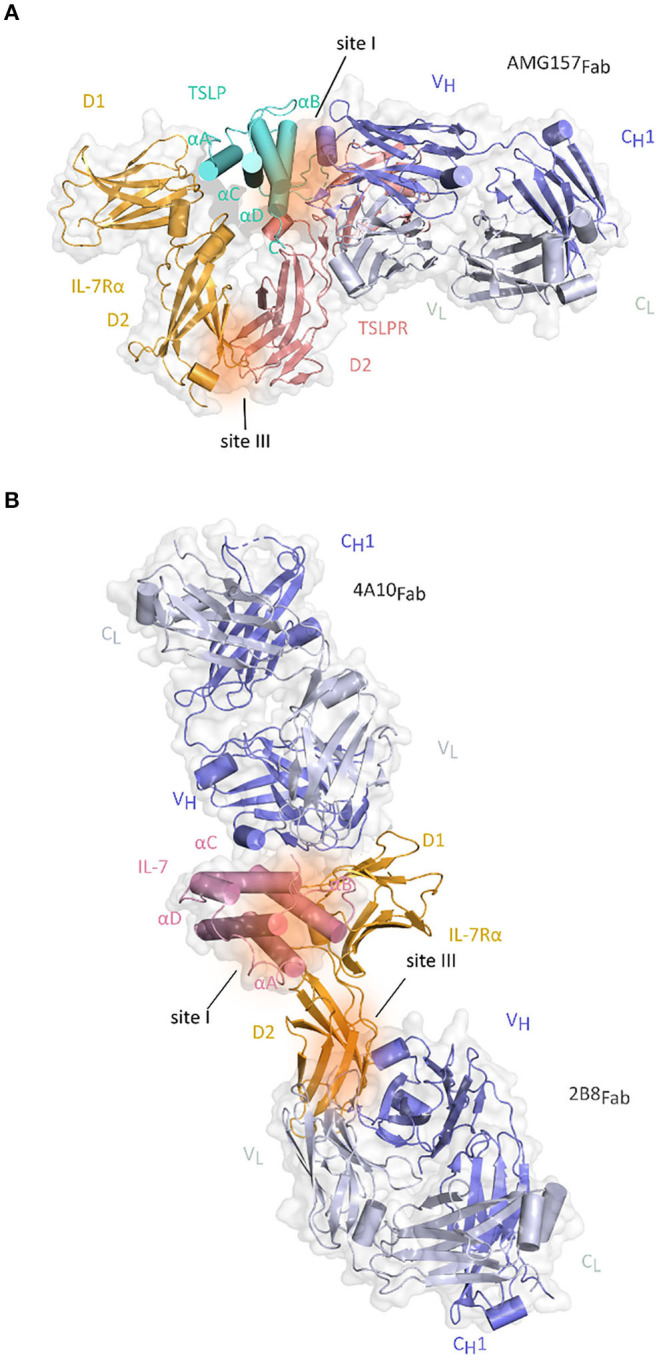
Structure of Fab fragments of the antibodies developed against TSLP and IL-7Rα together with their respective cytokine-receptor complexes. **(A)** Cartoon representation of the TSLP:TSLPR:IL-7Rα:AMG157_Fab_ complex by superposition of the TSLP:TSLPR:IL-7Rα and TSLP:AMG157_Fab_ based on structural alignment of the two TSLP structures [PDB ID:5J11, 5J13 (82)]. TSLP is shown in aquamarine cartoon representation with its four helices marked αA-αD. The extracellular regions of TSLPR (salmon pink) and IL-7Rα (bright orange) each comprising of two FnIII-like domains D1 and D2 are shown as cartoons on a transparent gray surface representation. Regions contributing to protein-protein interactions are represented by dark orange surfaces and named site I (TSLP:TSLPR) and site III (TSLPR:IL-7Rα). V_H_ and C_H1_ fragments of AMG157_Fab_ are colored in violet blue and the V_L_-C_L_ fragments in white blue and shown as cartoons on a transparent gray surface representation. **(B)** Cartoon representation of the IL-7:IL-7Rα:4A10_Fab_:2B8_Fab_ by superposition of IL-7Rα:4A10_Fab_ and IL-7Rα:2B8_Fab_ and IL-7:IL-7Rα based on structural alignment of IL-7Rα chains [PDB ID 3DI2, 6P50, 6P67 (99, 139)]. IL-7 is shown in pink cartoon representation with four helices marked αA-αD on a transparent gray surface representation. The two FnIII-like domains (D1 and D2) of IL-7Rα (bright orange) are shown as cartoons. Regions contributing to cytokine-receptor interactions are represented by dark orange surfaces and named site I for IL-7:IL-7Rα interface and site III for TSLPR:IL-7Rα and plausible γc interaction site. 4A10_Fab_ and 2B8_Fab_ are depicted as described for AMG157_Fab_ in **(A)**.

Anti-TSLP monoclonal antibody (mAb) Tezepelumab (AMG157/MEDI9929) has first been reported in 2014 and is to date the most prominent and advanced inhibitor of TSLP-mediated signaling in the context of allergic inflammatory disorders and the only TSLP-linked antagonistic candidate that is currently in phase III clinical trial in patients with severe asthma (136–138). The neutralizing effects of this antibody suggested that it recognizes an epitope in the domain of cytokine responsible for binding of TSLPR chain. Indeed, the X-ray structure of AMG157_Fab_ fragment in complex with TSLP confirmed this by showing that complementarity determining regions (CDRs) of the variable heavy chain domain of Fab fragment interact with the AB-loop region and C-terminal region of helix D, while the light chain fragment does not interact with TSLP. In the same time, the other side of TSLP helical bundle involved in binding IL-7Rα remains available (82) (Figure 4A).

The most recent publication covering phase II trial in adults with a history of asthma exacerbations and uncontrolled asthma reports the study being conducted by subcutaneous Tezepelumab application over 52 weeks. Patients who were previously receiving asthma controllers and received Tezepelumab therapy showed rate reductions of clinically significant asthma exacerbations of at least 62% compared to the patients receiving placebo independent of baseline eosinophil counts (140, 141). Ongoing phase III clinical trial includes further mechanistic and long-term safety trials with the focus on both adult and adolescent patients with severe asthma considering previous or current treatments with different combinations of asthma controller medications. Initial safety and preliminary clinical activity of Tezepelumab in AD patients were evaluated in phase I study and showed no immunogenicity and good tolerance (142, 143). Phase IIa study in patients with moderate to severe AD receiving the antibody subcutaneously in combination with topical corticosteroids showed improvements in severity of the disease yet they were not statistically significant (144, 145). A Phase IIb study is designed to evaluate the safety and efficacy of Tezepelumab as a monotherapy and adjunct therapy in patients with moderate to severe AD and is currently in the patient recruiting stages (146). Confirmed evidence of efficacy of this antibody in treatment of a possibly broad population of asthma patients together with potential therapeutic benefit in patients with AD puts Tezepelumab on the map of promising therapeutics in inflammatory and possibly other diseases as well. Furthermore, the antibody-based inhibitor CSJ117 has been developed in a form of an inhalable Fab antibody fragment against TSLP. It has been used in a recently completed Phase I clinical trial in asthma patients with mild atopic asthma to access the safety, tolerability, pharmacokinetics, and pharmacodynamics of the inhaled agent (147). Moreover, novel human single-chain variable fragment (scFv) was also developed against human TSLP and selected from a fully human antibody library. ScFv29 is shown to bind to human TSLP in competition with the TSLPR. As it can also bind to murine TSLP, *in vitro* experiments performed on mouse derived mDCs showed that ScFv29 reduces the maturation rate of DCs making it one of the potential neutralizing candidates of this system (148). With the aim of increasing the therapeutic effects in asthma patients that have been shown to co-express TSLP and IL-13 and contribute to the severity of the disease Venkataramani and colleagues have developed novel bispecific anti-TSLP/IL-13 antibodies that are either monovalent bispecific (Zweimabs) or bivalent bispecific (Doppelmabs) and can simultaneously inhibit the signaling of both cytokines (149, 150). Binding and functional data show stronger affinity of the bispecific antibodies in comparison to their parental monospecific ones and inhibitory effects in DCs assays demonstrating the potency of such dual targeting (151) (Table 1).

**Table 1:** Overview of the current modulators of TSLP signaling in preclinical studies and clinical trials.

**Signaling modulator**	**Target**	**Preclinical studies**	**Target disease**	**Clinical trials**	**References**
Human IgG2λ mAb Tezepelumab MEDI9929 AMG157	TSLP	BAF3-TSLPR cells Human blood DCs	Inflammatory allergic diseases	Phase I asthma Phase I asthma adolescents Phase I healthy subjects Japan Phase IIb asthma Phase II CASCADE Phase I AD Phase IIa ALLEVIAD Phase I/Phase II cat allergy Phase III NAVIGATOR Phase III PATH-HOME Phase I PATH-BRIDGE	(136, 137) (152) (153) (140, 141) (154) (142, 143) (144, 145) (155) (138) (156) (157)
mAb IgG2λ Fab fragment CSJ117	TSLP		Inflammatory allergic diseases	Phase I asthma	(147)
Human scFv ScFv29	TSLP	Mouse derived mDCs	Inflammatory allergic diseases		(148)
Bispecific mAbs Zweimabs and Doppelmabs	TSLP and IL-13	Human blood DCs	Inflammatory allergic diseases		(151)
Humanized IgG1 mAb RG7258	TSLPR	Human DCs Human mast cells Ascaris-sensitive cynomolgus monkey model	Inflammatory allergic diseases	Phase I asthma (discontinued)	(158, 159)
Human IgG1 mAb ASP7266	TSLPR	Human and monkey peripheral white blood cells, human mDCs, cynomolgus monkeys, ascaris-sensitive cynomolgus monkeys model	Inflammatory allergic diseases	Phase I asthma Japan (discontinued)	(160, 161)
mAb 1E10	TSLPR	BaF3-TSLPR cells TSLPR^+^ BCP-ALL LTCs	Leukemia BCP-ALL		(162, 163)
TSLP cytokine traps	TSLP	HEK293T cells, human blood DCs	Inflammatory allergic diseases		(82)
Small molecule Baicalein	TSLP	HMC-1 cells, HDM-induced mouse model of airway inflammation, OVA-induced mouse model of pulmonary eosinophilia	Inflammatory allergic diseases		(164)

The inhibitory mAb RG7258 considered as a potential therapeutic in allergic disorders has been developed against TSLPR. It cross reacts with the cynomolgus monkey TSLPR and inhibits TSLP-induced responses *in vitro*. Furthermore, in the ascaris-sensitive cynomolgus monkey model mAb RG7258 successfully reduced inflammation and bronchoconstriction (158). Another anti-TSLPRα antibody ASP7266 is able to inhibit TSLP signaling in peripheral white blood cells and mDC-mediated differentiation of naive CD4+ T cells into mature T cells *in vitro*. Intravenous administration to monkeys effectively blocked CCL17 mRNA expression in peripheral blood cells and suppressed skin allergic reactions in sensitized cynomolgus monkeys (160). However, phase I clinical trial in asthma patients in Japan has been discontinued for undisclosed reasons (161). One additional mAb generated against extracellular domain of TSLPR was reported as one out of two hybridoma clones that showed antagonistic properties toward TSLP without affecting IL-7/IL-7R signaling (162). A subsequent study by the same group tested the antagonistic potential of 1E10 clone in the context of ALL and showed that blocking of TSLPR represses proliferation and STAT activity in TSLPR^+^ BCP-ALL long term cultures (LTC) making it a potential therapeutic option for subset of BCP-ALL patients whose lymphoblasts express TSLPR (163) (Table 1).

The physiological role of soluble versions of cytokine receptor ectodomains as modulators of cytokine signaling in physiology and disease, and the fact that a naturally existing soluble TSLPR has not yet been identified, inspired the employment of the TSLPR ectodomain or its engineered variants as potential neutralizers of TSLP-mediated signaling. In a first study with such focus, a fusion protein TSLPR-Ig was designed by fusing the ectodomain of murine TSLPR with a murine IgG2a Fc tail. The effect of blocking TSLP signaling by murine TSLPR-Ig was tested in TSLP-activated murine DCs *in vitro* where it reduced the expression of CD40, CD80, and CD86. Additionally, local administration of murine TSLPR-Ig into the airways of asthmatic mice before sensitization suggested altering of the function of pulmonary DCs critical in Th2-mediated allergic disorders demonstrating that this blocking strategy could indeed be beneficial for treatment of asthma (165). Another validation of using TSLPR complex ectodomains as inhibitors was demonstrated by utilizing both soluble TSLPR and IL-7Rα ectodomains in an equimolar mixture in a cellular STAT5 activity assay confirming their dose-dependent neutralizing effect. Arguably, the most potent approach to date situates in the employment of receptor fusion proteins, termed TSLP-traps, featuring tandem fusions of the TSLPR, and IL-7Rα ectodomains using a flexible (Gly-Gly-Ser)_20_ linker. Notably, TSLP-trap1 showed a 250-fold higher affinity to TSLP when compared to the unlinked receptor ectodomains and a comparable affinity and binding kinetics to TSLP as both Tezepelumab mAb and its corresponding Fab fragment. A similar binding profile has been reported for TSLP-trap2. Competition assays in HEK293T cells showed that both TSLP-trap variants have about 1000-fold higher inhibitory potency over equimolar mixtures of unlinked soluble ectodomains and could outperform the inhibition of STAT5 signaling by 20–30-fold in comparison to the most potent anti-TSLP agent Tezepelumab and its Fab fragment. These intriguing data were further supported by evidence of both TSLP-trap variants having no effect on IL-7 function and being able to significantly block TSLP-driven DC-activation with the same efficacy as Tezepelumab (82). Thus, TSLP-traps represent promising novel biologicals with outstanding neutralizing potency.

The advent of high-resolution crystal structures of mouse and human TSLP:TSLPR ternary complexes (81, 82) offered the long missing structural blueprints to inspire the development of small molecule inhibitors targeting the TSLP:TSLPR interaction as a new therapeutic strategy. Van Rompaey and collaborators identified the first fragments to inhibit the TSLP:TSLPR interaction by a combined virtual—*in vitro* screening approach. The procedure consisted of an extensive *in silico* analysis of the structural data and evaluation of possible hot spots, screening of two commercially available fragment libraries followed by docking the hits to TSLPR, two-stage biological screening for further selection of the fragments and finally molecular dynamics to explore the binding pathway and model of fragment binding. This approach provided a proof-of-concept for the use of fragments in the modulation of TSLP signaling (166). Another study explored the potential of peptide-derived inhibitors designed based on amino acid sequences from murine TSLP:TSLPR structure that was the only structure available at the time. Solid-phase peptide synthesis was used to generate 16 peptides by mimicking epitopes of two TSLP:TSLPR interaction sites and resulted in three peptides capable of TSLP inhibition at submillimolar concentrations (167). The most recent report has focused on a flavonoid representing a major component of *S. baicalensis*, Balcalein, that was identified as the first small molecule inhibitor of TSLP-mediated signaling. Based on *in vitro* confirmation that the compound blocks TSLP:TSLPR interaction in a dose-dependent manner, *in vivo* studies in both HDM-challenged and OVA-sensitized mice resulted in reduced number of eosinophils in treated mice. Further chemical modeling led to a synthesis and identification of compound 11a, a biphenyl flavanone analog, which is considered the most advanced human TSLP inhibitor in this series of tests and characterized by moderate inhibition and good water solubility (164).

Efforts to identify TSLP antagonists for both murine and human cytokine have been made by analyzing TSLP:TSLPR and TSLP:IL-7Rα interaction sites. The most promising murine TSLP variant had one-point mutation, I37E, and showed high-affinity binding to TSLPR but no STAT5 activity in a cellular based assay (81, 82). From the selection of generated human mutants, a double mutant carrying S45R/T46R mutations residues at the TSLP:IL-7Rα interface in site II showed an unaffected ability to bind TSLPR and reduced STAT5-signaling in comparison to wt TSLP. From the selected mutants located in site I at the TSLP:TSLPR interface all of them reduced STAT5 signaling, with R149S/R150S double mutant having the most pronounced effect (82). In spite of not yielding a potent antagonist, those functional interrogations did demonstrate the importance of TSLP residues identifying hot spots that could be considered in further attempts in antagonist design.

## Modulators of IL-7 Signaling

Targeting of IL-7:IL-7Rα for therapeutic development in autoimmune diseases has mainly focused on the development of monoclonal anti-IL-7Rα antibodies which could be beneficial by blocking IL-7Rα and subsequently attenuating the action of effector T cells but retaining the T_reg_ activity (168).

Application of anti-IL-7Rα mAb 28G9/Ab1 in mice with autoimmune encephalomyelitis (EAE) was effective in reducing disease activity and severity (169). In non-obese diabetic mice, treatment with 28G9 delayed the progression of T1D before onset and reversed the newly onset diabetes (170). *Ex vivo* studies in cynomolgus monkeys showed a decreased STAT5 phosphorylation in both CD4+ and CD8+ T cells of blood samples after treatment (171). Another mAb (PF-06342674/RN168) developed by Pfizer has completed a phase I clinical trial in healthy volunteers. While the MS clinical trial was terminated by Pfizer themselves, the T1D phase I clinical trial evaluated the safety and tolerability of multiple SC doses in type 1 diabetes patients and showed that certain dose of mAb selectively inhibits survival and activity of memory T cells while preserving naive T cells and T_reg_ (172). Additional model study of its pharmacokinetics showed that it has a 20-fold more potent inhibitory effect on T_EM_ cells relative to T_reg_ cells at a similar dose confirming the implication that these effects could serve to eliminate pathologic T cells in autoimmune diseases (173) (Table 2).

**Table 2:** Overview of the current modulators of IL-7 signaling in preclinical studies and clinical trials.

**Signaling modulator**	**Target**	**Preclinical studies**	**Target disease**	**Clinical trials**	**References**
Human IgG1 mAb Ab1 28G9	IL-7Rα	Mice with EAE, mouse model T1D, cynomolgus monkeys	Autoimmune diseases		(169–171)
Humanized IgG1 mAb PF-06342674 RN168	IL-7Rα		Autoimmune diseases	Phase I healthy volunteers Phase Ib T1D (completed) Phase I MS (terminated)	(174) (172, 175) (176)
Humanized Fc-disabled mAb GSK2618960	IL-7Rα	No data publicly available (GSK)	Autoimmune diseases	Phase I healthy volunteers Phase I RRMS (terminated) Phase IIa pSS (withdrawn)	(177, 178) (179) (180)
Fully human IgG1 mAb B12 Antibody drug conjugate B12-MMAE	IL-7Rα	Ba/F3 and D1 cell lines, T-ALL cell lines, primary human T-ALL cells, NK-cells, Rag1–/– mice in combination with D1 cells and T-ALL cells	Leukemia (T-ALL)		(181)
Chimeric FAb human IgG1 4A10 2B8	IL-7Rα	D1 cell line, primary human T-ALL cells, Rag1–/– mice in combination with patient derived xenografts (PDX) cells	Leukemia (T-ALL)		(139)
rIL-7 CYT107		C57BL/6, c57BL/6-L5.1, BALB/c mice, CD1 mice	HIV, sepsis, Hematopoietic stem cell transplantation (HSCT), cancer	Phase I/II solid tumors Phase II sepsis IRIS-7-B Phase II cancer Phase I HSCT Phase I HIV Phase II HIV ERAMUNE-01 Phase I HIV INSPIRE Phase I cancer Phase II cancer ELYPSE-7 Phase I/II HCV ECLIPSE 1	(182) (183, 184) (185) (186, 187) (188, 189) (190, 191) (192) (193) (194, 195) (196) (197–199)
rIL-7 with hybrid human Fc IL-7-Fc NT-I7 GX-I7 Efineptakin alfa Hyleukin		Mice with syngeneic tumor graft, cynomolgus monkeys, BALB/c mice, C57BL/6 mice, and DO11.10 T cell receptor (TCR) transgenic mice, human colon adenocarcinoma xenograft mice	Cancer	Phase I healthy volunteers Phase I HPV	(200) (201) (202–204)

Humanized Fc-disabled anti-IL-7Rα mAb, known under GSK261896, was well-tolerated in phase I clinical trial in healthy volunteers. It blocked IL-7 receptor signaling upon full target engagement, increased circulating IL-7 and soluble IL-7Rα, however showed no impact on peripheral T cell subsets or levels of other inflammatory cytokines (177). Phase I clinical trial in relapsing remitting MS patients got terminated because of the misrepresentation of preclinical data while the phase II trial in pSS patients was withdrawn resulting in no ongoing trials for this agent at the moment (180) (Table 2).

Two of the most recently developed anti-IL-7Rα antibodies might both potentially benefit patients with T-ALL. Akkapeddi and colleagues reported a fully human IgG1 mAb, termed B12, developed against wildtype and several mutant IL-7Rα carrying insertions or single amino acid substitutions. A simulation of the structure of B12 in complex with the non-glycosylated IL-7Rα ectodomain indicated that the binding epitope is distinct from the interaction interface with IL-7. Based on the known structure of IL-7:IL-7Rα structure, IL-7 is positioned at the region connecting the D1 and D2 domains of the receptor, which implies that B12 could be located on the opposite side. B12:IL-7Rα binding interface is mostly hydrophobic with few van der Waals interactions and hydrogen bonds. B12 is able to inhibit IL-7-dependent and mutant-dependent IL-7R-mediated signaling and induce leukemia cell death. It promotes NK-mediated T-ALL cytotoxicity *in vitro*, delays T-cell leukemia development *in vivo* reducing tumor burden and promoting mouse survival and sensitizes T-ALL cells to treatment with dexamethasone inducing leukemia cell death. Those favorable effects were meliorated by B12-mono-methyl auristatin E (MMAE) antibody–drug conjugate (ADC) that is able to kill primary and patient-derived xenograft T-ALL cells more efficiently than B12 alone (181). The possibilities of ADCs in combination with IL-7R have previously been considered after confirming the involvement of IL-7 signaling in steroid-resistance when addressing the treatment of autoimmune diseases and cancers. The developed anti-murine IL-7R antibody conjugated with the compound SN38 showed strong anti-tumor effects against parental and steroid-resistant malignant cells, while the antibody-MMAE conjugate suppressed the inflammation in the mouse autoimmune arthritis model suggesting this approach as a possible novel alternative to steroid therapy (205). Furthermore, second study by the same group of collaborators that generated B12 shows that the two newly designed chimeric mAbs 4A10, and 2B8 recognize two separate epitopes on IL-7Rα based on the crystal structure of the 4A10_Fab_:IL-7Rα:2B8_Fab_ complex. This structure reveals that 4A10_Fab_ interacts with the periphery of epitopes responsible for binding IL-7 and that 2B8_Fab_ binds close to the membrane region of the IL-7Rα, where TSLPR and the predicted γc binding sites would be situated (Figure 4B). Binding of 4A10F_ab_ to the extracellular portion of IL-7Rα has been shown to be 9-fold tighter than the binding of 2B8F_ab_ with both *K*_D_ values in the low nM range compared to the binding properties of the mAbs to IL-7Rα on human lymphocytes. Moreover, these mAb chimeras inhibit IL-7R signaling at low IL-7 concentrations, mediate antibody-dependent cell mediated cytotoxicity *in vitro* and are effective in controlling established and relapsing leukemia *in vivo* (139) (Table 2).

Due to the pleiotropic nature of the biological activity of IL-7 and its central role in T-cell development, recombinant IL-7 (rIL-7) has been extensively tested in another, agonistic modulating frame. Indeed, multiple preclinical studies have confirmed that it could have therapeutic applications due to its potent immunorestorative and enhancing effect in immunotherapy and target multiple immunodeficiency conditions (206).

When used as an adjuvant in immunotherapy in sepsis patients with septic shock and severe lymphopenia, rIL-7 administered intramuscularly caused a 3- to 4-fold increase in absolute lymphocyte counts, reversed the marked loss of CD4+ and CD8+ immune effector cells and increased T cell proliferation and activation thus restoring adaptive immunity. As a novel approach in the treatment of patients with sepsis, another phase II study is planned in the future based on intravenous administration of rIL-7 (183, 184) (Table 2). Furthermore, patients suffering from infectious diseases may benefit from rIL-7 therapy given in a combination with antiviral drugs (188–192, 207), while patients who have received rIL-7 after hemopoietic stem cell transplantation (HSCT) showed T-cell recovery, implying a possibility of a lower risk of subsequent infection and relapse (186, 187) (Table 2).

In the context of cancer treatments rIL-7 has been used in clinical trials in patients with diverse types of tumors. A first study in humans was done in patients with metastatic melanoma and sarcoma and showed the ability of this cytokine to increase the number of CD4^+^ and CD8^+^ lymphocytes and decrease in the percentage of CD4^+^ T-regulatory cells suggesting its role in treating lymphopenia. At the same time, this study showed that the non-glycosylated variant of IL-7 elicits a low titer of binding antibodies and could lead to potential side effects in higher doses, suggesting the advantage of IL-7 produced in eukaryotic systems (197). A Phase I study in patients with solid tumors has been completed and proved tolerance and rIL-7 potency resulting in rejuvenated circulating T-cell profile with increase in overall naive T cells but a decreased T_reg_ number making this effect opposite from the one observed in treatments employing IL-2 (198). In lymphopenic metastatic breast cancer patients during Phase II trial it increased CD4+ and other T-cell subset counts without altering their function (194, 195). Combining rIL-7 with vaccine therapy was used in two completed phase I clinical trials in patients with melanoma and pediatric solid tumors (182, 193). A phase II trial in patients with prostate cancer used glycosylated rIL-7 after vaccine therapy (185) (Table 2). The glycosylated version of rIL-7 will also be used in Phase II study in patients with locally advanced bladder urothelial carcinoma in a combination with Atezolizumab, an anti-PD-L1 antibody (208). The phase II study using chimeric antigen T cell therapy (CAR-T) in treating malignant solid tumor is planned to use patient's T cells and engineer them into IL-7 and Chemokine (C-C Motif) Ligand 19-expressing CD19-CAR-T cells and transfuse them into the patient for treatment of their B cell lymphoma (209).

A fusion protein, IL-7-Fc, composed of a recombinant form of IL-7 and a hybrid Fc region of a human antibody has been shown to stimulate proliferation and survival of different T-cell subsets and enhance anti-tumor immune responses (202). A phase I study on healthy volunteers was completed with further clinical trials at the moment either recruiting patients or planned for treatments of different types of malignancies: high risk skin cancer treatment in combination with Atezolizumab, treatment of high-grade glioma, treatment in combination with cyclophosphamide in patients with solid tumors being some of them (200, 210–212). Another phase I study will test the effects of IL-7-Fc on enhancement of immune reconstitution and vaccine responses in older people following chemotherapy due to their weakened immune system (213). Fc-fused IL-7 could also be used for inducing humoral immunity against viruses and a phase I clinical trial in human papilloma virus infected patients has been completed (201, 203, 214). Additionally, preclinical studies imply that IL-7-Fc can be used as an adjuvant in DNA vaccines and improve the immunogenicity (215, 216) (Table 2).

## Perspectives and Conclusions

The landscape of therapeutic agents that can modulate the bioactivity of TSLP and IL-7 in inflammation, autoimmunity and cancer is clearly very broad in terms of disease coverage and displays a strong focus on biologics. Indeed, a number of therapeutic agents have either already entered the market or are in the final stages of clinical studies as demonstrated (217, 218). The diversity of agents that have been developed to block TSLP action demonstrates the range of possibilities and approaches that could be used to alter the biological activity of cytokines in general. Although most of the reported signal mediators of TSLP seem to be developed with the intention of treating inflammatory allergic diseases with Tezepelumab being the most promising novel therapeutic for asthma treatment, these agents should also be considered in the future in other, non-allergen induced conditions such as leukemia or autoimmune diseases. Precise dissection of the role of TSLP in each type of solid cancers will be key to enabling appropriate therapeutic strategies. For instance, in cancer types where TSLP might be tumor-protective, recombinant TSLP could prove to have therapeutic value either independently, or in the form of an immunocytokine fusion for tumor suppression by analogy to IL-2 (219, 220). Leveraging on the available structural data on the TSLP-receptor complex together with diverse display techniques to select hits with tailored characteristics could be considered in the design of TSLP antagonists or agonists (115, 221, 222).

Targeting upstream signaling mechanisms by different therapeutic approaches is considered to be potentially beneficial in preventing relapse and maintaining remission in patients with chronic inflammatory disorders or autoimmune diseases (223). For instance, this is supported by evidence that blocking the IL-7 mediated pathway can reverse ongoing autoimmunity (224). In the context of autoimmune diseases and cancer, the IL-7 signaling axis has been extensively targeted with antibodies against IL-7Rα which is rational considering that the effect of blocking γc chain could be problematic and lead to severe side effects since it is shared with numerous other cytokines (225). This approach has led to the identification of neutralizing antibodies that have completed phase I clinical trials and could potentially become beneficial for patients with autoimmune diseases. An additional reason for addressing IL-7Rα in drug development are frequent mutations leading to homodimerization of the receptor chains and constitutive signal transduction in a high percentage of B-ALL and T-ALL patients.

Considering the important role of both TSLP and IL-7 in the pathogenesis of RA simultaneous inhibition of both TSLP and IL-7R signaling in arthritis could serve a plausible therapeutic rationale in arthritis (226).

Although characterized by high selectivity, high efficacy, and limited side effects, biologics generally face a number of challenges such as expensive production, low tissue penetration and invasive administration (227–229). The design of small molecule inhibitors, an approach that highlights the importance of available structural information for facilitation of the design processes could present a suitable parallel alternative successfully addressing some of those issues. Ongoing and future studies on the diverse roles of TSLP and IL-7 in physiology and disease will undoubtedly further fuel efforts in the targeting of the two pleiotropic cytokines and their receptors in autoimmune diseases and cancer via appropriate molecular modulators.

## Author Contributions

IM and SS designed the scope and thematic coverage of the article. IM reviewed the current literature, wrote the main draft of manuscript, and generated the figures. SS reviewed the drafts, provided conceptual and textual input, and approved the final version of the manuscript. Both authors contributed to the article and approved the submitted version.

## Conflict of Interest

SS is listed as co-inventor in a patent application PCT/EP2017/057944 focusing on the development and application of TSLP-traps. The remaining author declares that the research was conducted in the absence of any commercial or financial relationships that could be construed as a potential conflict of interest.
